# Amyotrophic lateral sclerosis care in Saudi Arabia: A survey of providers’ perceptions

**DOI:** 10.1002/brb3.1795

**Published:** 2020-08-15

**Authors:** Ahmad R. Abuzinadah, Aysha A. AlShareef, Abdullah AlKutbi, Ahmed K. Bamaga, Ali Alshehri, Hussein Algahtani, Edward Cupler, Mohammed H. Alanazy

**Affiliations:** ^1^ Neuroscience Unit Neurology Division Internal Medicine Department Faculty of Medicine King Abdulaziz University Hospital King Abdulaziz University Jeddah Saudi Arabia; ^2^ Neuromuscular Unit King Fahad Medical Research Center King Abdulaziz University Jeddah Saudi Arabia; ^3^ Neurology Division Internal Medicine Department Faculty of Medicine King Abdulaziz University Hospital King Abdulaziz University Jeddah Saudi Arabia; ^4^ Neurology Department International Medical Center Jeddah Saudi Arabia; ^5^ Pediatric Department Faculty of Medicine King Abdulaziz University Hospital King Abdulaziz University Jeddah Saudi Arabia; ^6^ Neurosciences Department King Faisal Specialist Hospital and Research Center Riyadh Saudi Arabia; ^7^ Neurology Department King Abdulaziz Medical City National Guard Hospital King Saud bin Abdulaziz University for Health Sciences Riyadh Saudi Arabia; ^8^ Neurosciences Department King Faisal Specialist Hospital and Research Center Jeddah Saudi Arabia; ^9^ Department of Internal Medicine King Saud University Medical City King Saud University Riyadh Saudi Arabia

**Keywords:** amyotrophic lateral sclerosis, care, multidisciplinary, Saudi Arabia

## Abstract

**Objective:**

Provision of care for patients with amyotrophic lateral sclerosis (ALS) is complex and requires the contribution of multiple healthcare professionals. Several international ALS care measures were developed to ensure optimal care for ALS patients. We looked at the rate of inconsistency in providing standard ALS care measures in Saudi Arabia (SA).

**Methods:**

A 5‐point response survey was distributed to practicing neurologists in SA. They were asked to grade their perceived consistency of accessibility for 19 items of ALS care measures at their center. The list of ALS care measures items was derived from international ALS guidelines.

**Results:**

The response rate from neurologists was 47.3% (62/131), and the responses of 39 neurologists who follow ALS cases were included. Most of the selected ALS care measure items, 63.1% (12/19), were perceived by 50% or more of the ALS care providers to be not consistently accessible to their patients. The perception of ALS care providers of the inconsistent accessibility for ALS patients to ALS care measures was high for communication devices (92.3%), supportive equipment such as motorized wheelchairs (76.9%), end‐of‐life discussion (74.4%), and respiratory monitoring (66.7%).

**Conclusion:**

Our data show that ALS patients in SA do not have consistent access to the recommended ALS care measures.

## Graphical abstract

Amyotrophic lateral sclerosis (ALS) is a progressive neurodegenerative disease that requires a coordinated health care measures in order to improve patient's survival and quality of life as well as to reduce caregiver burn out. We conducted a survey for ALS care providers and we found that there is a low accessibility rate to standard ALS care measures that were recommended per international guidelines.

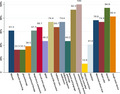

## 1 INTRODUCTION

Amyotrophic lateral sclerosis (ALS) is a neurodegenerative disease that affects the upper and lower motor neurons, resulting in motor disability, bulbar dysfunction, respiratory failure, and early mortality. The prevalence of ALS is approximately 5–10 people per 100,000 population, with an incidence of 2 per 100,000 population per year (Benjaminsen, Alstadhaug, Gulsvik, Baloch, & Odeh, 2018; Doi, Atsuta, Sobue, Morita, & Nakano, 2014; Logroscino & Piccininni, 2019; Mehta et al., 2018). However, the lifetime risk of developing ALS is 1 in 350–420 (Armon, 2007). The disease results in the inability of the patient to perform activities of daily living such as walking, feeding, and self‐care. This leads to an economic impact on the patients, their families, and societies (Gladman & Zinman, 2015). Hospitalization of ALS patients is associated with expensive costs (Cordesse, Sidorok, Schimmel, Holstein, & Meininger, 2015; Lechtzin, Wiener, Clawson, Chaudhry, & Diette, 2001). The cost can be reduced, with a higher quality‐of‐life for patients, with specialized care (Zwicker et al., 2019).

Several guidelines have been developed in order to ensure high quality of care. These guidelines include ALS care measures that improve quality‐of‐life and survival (Andersen et al., 2005, 2012; Miller et al., 2009a, 2009b). However, adherence to and utilization of these guidelines are poor (Miller, Anderson, et al., 2009). Quality improvement measures, as well as database tools, have been developed in order to improve adherence to guidelines for ALS care (Miller, Anderson, et al., 2009; Miller et al., 2014). These measures can also be used to assess the quality of care provided to ALS patients in a given area.

Adhering to guidelines should aid in recognizing and preventing patients from undergoing unnecessary suffering, and subsequently reduces the emotional and financial impact on the patients and their caregivers, as well as reducing the economic impact on the healthcare system. Our study aimed to explore the gaps in care and the degree of accessibility and utilization of the guidelines of ALS care measures, through surveying neurologists who manage ALS patients in Saudi Arabia.

## 2 Method

We created a questionnaire based on a 5‐point frequency scale ranging from “never” to “always” and confidence scale ranging from “not confident” to “extremely confident”, with a few items using “yes” or “no” answers, as appropriate. The questionnaire items were derived from the published guidelines of ALS management (Miller et al., 2009a, 2009b). Four neuromuscular neurologists were asked to independently review the provisional version of the survey in order to ensure content validity and clarity, and their input was incorporated into the final version (Supporting information). The survey was designed (using https://surveymonkey.com) and distributed to participants through email and private messages during the months of October 2018 to February 2019. We included all board‐certified neurologists who are registered with the Saudi Commission for Health Specialties (SCFHS), including neuromuscular neurologists and general neurologists. Two additional reminders were sent to nonresponders two months apart. The study was approved by the institutional review board of King Abdulaziz University. The participants provided their consent at the beginning of the survey.

### 2.1 Survey reliability and validity

The internal consistency of the survey was evaluated by using Cronbach *α* coefficients. The content validity was achieved mainly through agreement between four neuromuscular specialists on the survey content. Additionally, the validity was evaluated through exploratory factor analysis. Cronbach *α* > 0.8 and appropriate factor loading (eigenvalues > 1) explaining >50% of the variance were considered sufficient.

### 2.2 Objectives

The primary objective was to identify the ALS care measure items that are perceived by ALS care providers to be inconsistently available to ALS patients. The secondary objective was to compare the ALS care measure accessibility between different cities in Saudi Arabia (the cities of Jeddah and Riyadh as the two large metropolitan areas, and other cities). We investigated the wait time to receive timely needed services such as bi‐level positive airway pressure ventilators (BiPAP) and percutaneous endoscopic gastrostomy (PEG) tube insertion. Additionally, we looked at the confidence and comfort level among ALS care providers in certain aspects of ALS care.

### 2.3 Statistical analysis

The demographics of the participants were described using frequencies. The responses of participants regarding their perceived frequency of access to ALS care measure items were dichotomized into inconsistent (never, rarely, and sometimes) and consistent (often and always). Similarly, the confidence level was dichotomized into confident (very confident and extremely confident) and suboptimal confidence (not confident, not so confident, and somewhat confident). The chi‐square test was employed to assess whether the provision of ALS care measure items was different across different cities.

## 3 RESULTS

The questionnaire was sent to 131 neurologists from 14 cities in SA; 62 of them (47.3%) responded. There were 36 (58.1%) neurologists practicing in tertiary hospitals, 17 (27.4%) in secondary hospitals, and 9 (14.5%) in private hospitals. There were 43 (32.8%) neurologists who follow at least one ALS case per year. Of these, four did not finish the survey; therefore, the responses from only 39 (29.7%) neurologists were included in the analysis (referred to as ALS care providers hereafter). There were 23 (37.1%) neurologists from Jeddah, 13 (20.9%) from Riyadh, and the remainder were from other cities (Table 1).

**Table 1:** Participants’ demographics (*N* = 43)

	%
Male	72.1
Age	
30–39 years	39.5
40–49 years	46.5
50–59 years	6.9
60–69 years	6.9
70 years or more	0
Subspecialty	
General neurologists	23.3
Stroke	16.3
Epilepsy	13.9
Multiple sclerosis	11.6
Neuromuscular	25.6
Cognitive and Dementia	2.3
Movement disorders	4.7
Clinical neurophysiology	2.3
City	
Jeddah	39.5
Makkah	9.3
Riyadh	25.6
Al‐Hafoof	2.3
Dammam	4.7
Al‐Khubar	2.3
Taboul	2.3
Al‐Baha	2.3
Al‐Madinah	4.7
Al‐Dhahran	4.7
Abha	2.3
Number of ALS cases per year (include all respondents, *n* = 62)	
Less than one case	30.7
1–5 cases	53.2
6–10 cases	8.1
11–15 cases	4.8
16–20 cases	3.2

*N* = 43 participants, (43 neurologists who follow at least one ALS case per year and 39 neurologists who completed the survey).

### 3.1 Survey reliability and validity

Cronbach *α* was 0.88 which indicated a satisfactory reliability. Four neuromuscular specialists agreed on the survey content indicating satisfactory content validity. Exploratory factor analysis revealed six factors with eigenvalues >1 and explaining 75% of the variance (Tables S1 and S2).

### 3.2 Items of ALS care perceived to be not consistently accessible by ALS care providers

**Figure 1:**
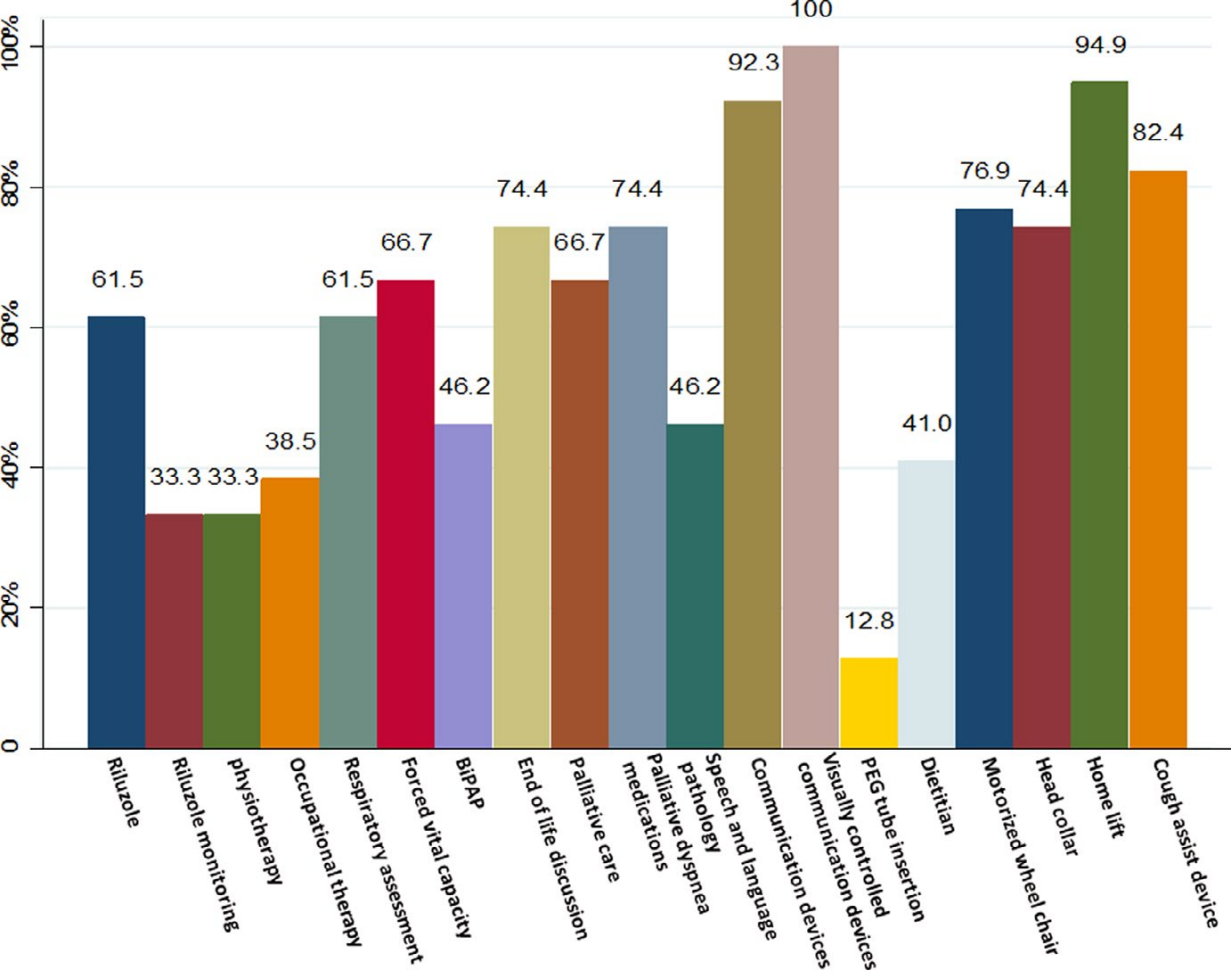
Proportion of ALS care providers perceived items of ALS care to be not consistently accessible. BiPAP, bi‐level positive airway pressure ventilators; PEG, percutaneous endoscopic gastrostomy

Twelve out of the 19 items included in the questionnaire were perceived by 50% or more of the ALS care providers to be not consistently accessible to their patients (Figure 1). These items were riluzole, respiratory and vital capacity assessment every 3 months, end‐of‐life discussion, palliative care, communication devices, visually controlled communication devices, motorized wheelchairs, head collars, home lifts, and cough assist devices. There were additional six items perceived by 33%–49% of the ALS care providers to be not consistently accessible to their patients, which included monitoring of riluzole blood work, physiotherapy access, occupational therapy access, BiPAP access, speech and language pathology access, and dietitian access.

There were no differences between different cities in accessibility to items of ALS care measures, except for more accessibility to motorized wheelchairs in Riyadh and other cities, as compared to Jeddah (Table 2).

**Table 2:** Frequency of inconsistently accessing items of care for ALS patients as perceived by ALS care providers

Item of ALS care	Frequency of inconsistently accessing the item of care	Jeddah	Riyadh	Others	*p* value
Riluzole access % (*n*/*N*)	61.5 (24/39)	68.8 (11/16)	55.6 (5/9)	57.1 (8/14)	.74
Monitoring Riluzole % (*n*/*N*)	33.33 (13/39)	43.8 (7/16)	11.1 (1/9)	35.7 (5/14)	.25
Physiotherapy access % (*n*/*N*)	33.33 (13/39)	31.3 (5/16)	55.6 (5/9)	21.4 (3/14)	.232
Occupational therapy access % (*n*/*N*)	38.5 (15/39)	43.8 (7/16)	11.1 (1/8)	50 (7/14)	.148
Respiratory assessment every 3 months % (*n*/*N*)	61.5 (24/39)	56.3 (9/16)	55.6 (5/9)	71.4 (10/14)	.636
Forced vital capacity (FVC) measured every 3 months % (*n*/*N*)	66.7 (26/39)	62.5 (10/16)	66.7 (6/9)	71.4 (10/14)	.875
BiBAP access % (*n*/*N*)	46.2 (18/39)	43.8 (7/16)	22.2 (2/9)	64.3 (9/14)	.138
End‐of‐life discussion % (*n*/*N*)	74.4 (29/39)	75 (12/16)	66.7 (6/9)	78.6 (11/14)	.813
Palliative care access % (*n*/*N*)	66.7 (26/39)	50 (8/16)	77.8 (7/9)	78.6 (11/14)	.183
Palliative medications for dyspnea % (*n*/*N*)	74.4 (29/39)	68.8 (11/16)	66.7 (6/9)	85.7 (12/14)	.475
Speech and language pathologist access % (*n*/*N*)	46.2 (18/39)	50 (8/16)	44.4 (4/9)	42.9 (6/14)	.92
Communication device access % (*n*/*N*)	92.3 (36/39)	93.8 (15/16)	100 (9/9)	85.7 (12/14)	.437
Visually controlled communication device % (*n*/*N*)	100 (39/39)	100 (16/16)	100 (9/9)	100 (14/14)	–
Access to PEG tube insertion % (*n*/*N*)	12.8 (5/39)	12.5 (2/16)	11.1 (1/8)	14.3 (2/14)	.974
Dietitian access % (*n*/*N*)	41.0 (16/39)	31.3 (5/16)	44.4 (4/9)	50 (7/14)	.565
Motorized wheelchair access % (*n*/*N*)	76.9 (30/39)	56.3 (9/16)	88.9 (8/9)	92.9 (13/14)	.037
Access to appropriate head collar % (*n*/*N*)	74.4 (29/39)	75 (12/16)	88.9 (8/9)	64.3 (9/14)	.418
Home lift access % (*n*/*N*)	94.9 (37/39)	100 (16/16)	100 (9/9)	85.7 (12/14)	.152
Cough assist access % (*n*/*N*)	82.4 (28/34)	72.7 (8/11)	77.8 (7/9)	92.8 (13/14)	.388

Abbreviations: BiPAP, bi‐level positive airway pressure ventilators; FVC, forced vital capacity; PEG, percutaneous endoscopic gastrostomy.

### 3.3 Wait time to access items of care

Among the ALS care providers (*n* = 39), 35.9% reported access to BiPAP within 4 weeks or less, 17.9% reported 1–2 months, 15.4% reported 2–4 months, 15.5% reported 4–8 months, and 15.4% reported >8 months.

The time required to arrange for PEG tube insertion was reported to be 4 weeks or less by 79.5% of ALS care providers, 1–2 months by 15.4%, and 2–4 months by 5.1%.

Confidence and comfort levels among ALS care providers in certain aspects of ALS care are detailed in Table 3.

**Table 3:** Suboptimal confidence and comfort levels in ALS care among all participants (*n* = 39)

	ALS care providers %
Comfortable sometimes or less in making ALS diagnosis	11.6
Comfortable sometimes or less in breaking ALS diagnosis to the patient	23.3
Confident somewhat or less to discuss the benefits of Edaravone	74.4
Confident somewhat or less to answer your patients' questions regarding stem cell transplantation	48.7
Confident somewhat or less in discussing end‐of‐life subjects	66.7
Confident somewhat or less to discuss invasive ventilation and tracheostomy	51.3

A minority of ALS providers feel uncomfortable with making an ALS diagnosis (11.6%); however, almost a quarter (23.3%) of ALS care providers feel uncomfortable breaking the news of an ALS diagnosis.

Many ALS care providers feel suboptimal confident in discussing the use of edaravone (74.4%), stem cell transplantation (48.7%), end‐of‐life discussion (66.7%), and tracheostomy and invasive ventilation (51.3%).

## 4 DISCUSSION

Our study shows that more than half of ALS care measures are provided inconsistently to ALS patients in Saudi Arabia. An important factor related to this low accessibility is the lack of multidisciplinary clinics in which ALS care can be coordinated. As ALS progresses, it affects multiple systems that require specialized healthcare professionals to address. Timely coordination between healthcare professionals reduces the rate of hospitalization of ALS patients from 79% to 37% (Cordesse et al., 2015). The lack of multidisciplinary clinics makes such coordination slower; this is supported by our data, especially in the delay in access to BiPAP, for instance.

One of the very important aspects of ALS care is end‐of‐life discussion. Unfortunately, this was perceived by 75% of ALS care providers as not done consistently. Three factors may explain this. First, lack of a timely input from other care providers (multidisciplinary clinic) to allow a more accurate prognostication, and more comfort and support to the care provider when discussing end‐of‐life issues with patients (Hogden, Greenfield, Nugus, & Kiernan, 2012). The second reason is the suboptimal confidence of the care providers in conducting these discussions. For example, almost two‐thirds of the neurologists involved in ALS care feel somewhat confident or less in having the end‐of‐life discussion. The importance of end‐of‐life discussion includes maintenance of patients’ and caregivers’ quality‐of‐life in parallel with avoidance of futile interventions (Connolly, Galvin, & Hardiman, 2015). In addition, end‐of‐life discussion alleviates unnecessary fear and provides reassurance and support to ensure patients’ comfort and decrease caregiver burden (Connolly et al., 2015). And finally, the lack of palliative care programs that reduce end‐of‐life suffering. Our data shows lack of consistent accessibility to palliative care program in two‐thirds of ALS care providers’ centers. The presence of multidisciplinary care clinics for ALS will also help in training physicians and healthcare professionals involved in this process to be able to undertake it with less discomfort, and to accumulate experience in having such discussions. The use of relevant questionnaires to the patients and caregivers could make the process more standardized and subsequently increase the comfort level and quality of care (Bacci et al., 2016; Tarvonen‐Schröder, Kaljonen, & Laimi, 2018; Terada et al., 2017). For example, Ozanne et al showed central nervous system tumors patients are more likely to have pain assessment through validated tool which could possibility explain the better pain control they receive than ALS patients (Ozanne et al., 2019).

Another major deficit in ALS care in SA is the lack of provision of communication devices. Our data indicate that speech and language assessment is perceived to be undertaken inconsistently by almost half of ALS care providers, and the accessibility to communication devices was perceived to be inconsistent by 92%, whereas advanced devices such as visually controlled/eye‐tracking communication devices are not accessible at all (100%). The European Federation of Neurological Science recommends evaluation of speech every 3 months and provision of communication devices accordingly (Andersen et al., 2005). Early utilization of communication devices improves or at least stabilizes the quality‐of‐life and mood in ALS patients (Korner et al., 2013). In addition, visually controlled/eye‐tracking communication devices have been shown to improve quality‐of‐life and reduce depression and anxiety, in comparison to other alternatives (Caligari, Godi, Guglielmetti, Franchignoni, & Nardone, 2013).

Several respiratory care measures recommendations were established in order to improve quality‐of‐life and slow the disease progression (Andersen et al., 2005; Khairoalsindi & Abuzinadah, 2018; Miller et al., 2009a). Monitoring respiratory function with forced vital capacity (FVC) should be performed every 3 months; however, our data show that this is perceived to be done inconsistently by approximately two‐thirds of ALS care providers. Noninvasive ventilation, such as BiPAP, should be considered early, with onset of signs or symptoms of respiratory insufficiency (Bourke et al., 2006). The perceived inconsistency of accessing BiPAP in our survey was 46% and two‐thirds estimated the time of BiPAP accessibility to be more than 4 weeks, while 46% of the respondents estimated the time of accessibility to be more than 8 weeks. It has been suggested that an early start of NIV increases the rate of survival at three years by three times (43% with early starting of NIV and 14% with late start) (Vitacca et al., 2018). Dyspnea among ALS patients is a common cause of suffering that can be managed by NIV and, at a late stage, by palliative medications; however, provision of such medications was perceived to be low in this study. Other parameters of respiratory care, such as cough assist device, despite the uncertain implications on the disease, it should be considered for palliative reasons. The accessibility to cough assist device is inconsistent as perceived by the majority of ALS care providers.

Our study has several limitations including the small sample size, which is mainly due to the small number of specialized ALS care providers in Saudi Arabia. However, our sample included most neurologists taking care of ALS patients across all major cities and hospitals in Saudi Arabia (Tables S3 and S4). These results were not validated with the patients, perspectives regarding the care they receive. Our survey did not include several others ALS care measures, which we elected to omit to make responses to the survey more feasible and accurate. We did not assess the rate of hospitalization and intensive care unit admissions as a consequence of the suboptimal provision of the standard ALS care measures in Saudi Arabia. Differences in cultural preferences in regards to discussing ALS diagnosis, prognosis, health‐related issues, decision‐making, and goals of care were not explored in this study. However, our study, despite its limitations, highlights several areas that necessitate an immediate action by the stakeholders to improve the care of ALS patients to the level of the recommended standards of care as per international guidelines.

## 5 CONCLUSION

Our study revealed that ALS patients in SA do not have consistent access to the recommended ALS care measures. Despite our study was limited to SA, we believe that other regional countries may share a similar situation. ALS patients are a vulnerable group, and their quality‐of‐life could be less if their treatment is not delivered in a coordinated way. There is a huge need to advocate for this group of patients in order for them to receive their appropriate care. We believe that the first step to make such improvements is to implement multidisciplinary clinics, where most of the healthcare providers are available to meet the patient needs and achieve consensus regarding the optimum treatment options for each patient.

## CONFLICT OF INTEREST

Abuzinadah, AlShareef, AlKutbi, Bamaga, Alshehri, Algahtani, Cupler, and Alanazy report no disclosures.

## AUTHOR CONTRIBUTIONS

Ahmad R. Abuzinadah involved in study design, data acquisition and interpretation, statistical analysis, and writing the manuscript. Aysha AlShareef involved in study design, data acquisition, review of the pilot form, and reviewing the manuscript. Abdullah AlKutbi involved in writing and reviewing the manuscript. Ahmed Bamaga, Hussein Algahtani, and Edward Cupler performed data interpretation and reviewing the manuscript. Ali Alshehri and Mohammad H. Alanazy involved in study design, data acquisition, reviewing the pilot form, and reviewing the manuscript.

## Supporting information

Table S1‐S4

## Data Availability

All data will be available up on request to the corresponding author.
