# Supplementary material for: Amyotrophic lateral sclerosis care in Saudi Arabia: A survey of providers’ perceptions
Source: Brain Behav. 2020 Aug 15;10(10):e01795. doi: 10.1002/brb3.1795 (PMC7559620; doi:10.1002/brb3.1795)
Supplement: Supplementary file 1 — Table S1‐S4 [file BRB3-10-e01795-s001.docx]

Supplementary material for **Amyotrophic Lateral Sclerosis Care in Saudi Arabia: A Survey of Providers’ Perceptions**

**Reliability Data:**

**eTable 1: Cronbach’s alpha**

| **Reliability Statistics** | | |
| --- | --- | --- |
| Cronbach's Alpha | Cronbach's Alpha Based on Standardized Items | N of Items |
| .881 | .888 | 19 |

| **Item-Total Statistics** | | | | | |
| --- | --- | --- | --- | --- | --- |
|  | Scale Mean if Item Deleted | Scale Variance if Item Deleted | Corrected Item-Total Correlation | Squared Multiple Correlation | Cronbach's Alpha if Item Deleted |
| Riluzole access | 47.9677 | 108.699 | .618 | .797 | .870 |
| Monitoring Riluzole | 47.2581 | 114.065 | .460 | .554 | .876 |
| Physiotherapy access | 47.6129 | 122.378 | .149 | .367 | .886 |
| Occupational therapy access | 50.5484 | 119.523 | .627 | .815 | .875 |
| Respiratory assessment every 3 months | 47.9355 | 109.862 | .715 | .942 | .867 |
| Forced vital capacity (FVC) measured every 3 months | 48.0968 | 110.357 | .690 | .953 | .868 |
| BiBAP access | 47.7742 | 108.781 | .640 | .841 | .869 |
| End of life | 48.3871 | 111.712 | .443 | .709 | .878 |
| Palliative care access | 50.8387 | 120.606 | .575 | .925 | .877 |
| Palliative medications for dyspnea | 48.2258 | 113.381 | .480 | .738 | .876 |
| Speech and language pathologist access | 47.8065 | 110.028 | .522 | .667 | .874 |
| Communication device access | 49.1613 | 112.673 | .600 | .893 | .871 |
| Visually controlled communication | 49.6774 | 121.826 | .270 | .756 | .881 |
| Access to PEG tube insertion | 46.6774 | 118.959 | .424 | .681 | .877 |
| Dietitian access | 47.2903 | 118.546 | .416 | .790 | .877 |
| Motorized wheelchair access | 48.7097 | 109.680 | .569 | .825 | .872 |
| Access to appropriate head collar | 48.5161 | 114.391 | .461 | .766 | .876 |
| Home lift access | 49.0968 | 115.357 | .489 | .878 | .875 |
| Cough assist access | 48.7419 | 113.198 | .550 | .882 | .873 |

**Validity Data:**

**eTable 2: factor analysis**

| **Rotated Component Matrix^a^**  Factor 1:  Available items of ALS care but not need coordination to be implemented  Factor 2:  Available in limited places  Factor 3:  Not available items of ALS care related to essential communication and mobility  Factor 4:  Not available items of ALS care  Factor 5:  End of life related items of ALS care  Factor 6:  Available items of ALS care | | | | | | |
| --- | --- | --- | --- | --- | --- | --- |
|  | Component | | | | | |
|  | 1 | 2 | 3 | 4 | 5 | 6 |
| Riluzole access | .157 | .693 | .151 | .367 | .157 | -.118 |
| Monitoring Riluzole | .274 | .671 | -.248 | .264 | .061 | -.029 |
| Physiotherapy access | .029 | -.063 | .101 | -.021 | .056 | .883 |
| Occupational therapy access | .528 | .484 | .032 | .036 | .336 | .176 |
| Respiratory assessment every 3 months | .793 | .153 | .139 | .291 | .297 | .092 |
| Forced vital capacity (FVC) measured every 3 months | .864 | .117 | .211 | .302 | .076 | .027 |
| BiBAP access | .434 | .638 | .231 | .083 | .133 | -.054 |
| End of life | .215 | .229 | .099 | .120 | .702 | -.029 |
| Palliative care access | .559 | .111 | .416 | -.066 | .527 | .019 |
| Palliative medications for dyspnea | .111 | .126 | .061 | .403 | .601 | .382 |
| Speech and language pathologist access | .063 | .764 | .236 | -.073 | .114 | .305 |
| Communication device access | .283 | .105 | .827 | .289 | -.008 | .092 |
| Visually controlled communication | .043 | -.067 | .836 | -.082 | .166 | -.022 |
| Access to PEG tube insertion | .235 | .471 | -.196 | .118 | .008 | .611 |
| Dietitian access | .716 | .378 | -.132 | -.165 | -.002 | .096 |
| Motorized wheelchair access | .400 | .248 | .501 | .425 | -.432 | .205 |
| Access to appropriate head collar | .178 | -.051 | .462 | .714 | .210 | -.163 |
| Home lift access | -.122 | .333 | .614 | .384 | .060 | -.008 |
| Cough assist access | .110 | .278 | .026 | .824 | .094 | .125 |
| Extraction Method: Principal Component Analysis.  Rotation Method: Varimax with Kaiser Normalization. | | | | | | |
| a. Rotation converged in 8 iterations. | | | | | | |

**eTable - 3**

**eTable - 4**
